# Can we improve time to patency with vasoepididymostomy with an innovative epididymal occlusion stitch?

**DOI:** 10.1590/S1677-5538.IBJU.2024.0222

**Published:** 2024-05-20

**Authors:** Francesco Costantini Mesquita, Luis Felipe Savio, David Velasquez, Alexandra Varnum, Rodrigo Barros, David Miller, Francis Petrella, Ranjith Ramasamy

**Affiliations:** 1 University of Miami Desai Sethi Urology Institute Coral Gables Florida United States University of Miami - Desai Sethi Urology Institute, Coral Gables, Florida, United States;; 2 Departamento de Urologia Beneficência Portuguesa de São Paulo São Paulo SP Brasil Departamento de Urologia - Beneficência Portuguesa de São Paulo, São Paulo, SP, Brasil;; 3 University of Miami Miller School of Medicine Miami Florida United States University of Miami Miller School of Medicine, Miami, Florida, United States;; 4 Universidade Federal Fluminense Niteroi RJ Brasil Universidade Federal Fluminense - UFF, Niteroi, RJ, Brasil

## Abstract

**Introduction:**

Obstructive azoospermia occurs when there is a blockage in the male reproductive tract, leading to a complete absence of sperm in the ejaculate. It constitutes around 40% of all cases of azoospermia (1, 2). Blockages in the male reproductive tract can arise from either congenital or acquired factors, affecting various segments such as the epididymis, vas deferens, and ejaculatory ducts (3). Examples of congenital causes encompass conditions like congenital bilateral absence of the vas deferens and unexplained epididymal blockages (4). Acquired instances of obstructive azoospermia may result from factors like vasectomy, infections, trauma, or unintentional injuries caused by medical procedures (5). This complex condition affecting male fertility, presents two main treatment options: microsurgical reconstruction and surgical extraction of sperm followed by in vitro fertilization (IVF). Microsurgical reconstruction proves to be the most cost-effective option for treating obstructive azoospermia when compared with assisted reproductive techniques (6, 7). However, success rates of reconstruction defined by patency are as high as 99% for vasovasostomy (VV) but decline to around 65% if vasoepididymostomy (VE) is required (8, 9). Thus, continued refinement in technique is necessary in order to attempt to improve patency for patients undergoing VE. In this video, we show a comprehensive demonstration of microsurgical VE, highlighting the innovative epididymal occlusion stitch. The goal of this innovative surgical technique is to improve outcomes for VE.

**Materials and Methods:**

The patient is a 39-year-old male diagnosed with obstructive azoospermia who presents for surgical reconstruction via VE. His partner is a 37-years-old female with regular menstrual cycles. The comprehensive clinical data encompasses a range of factors, including FSH levels, results from semen analysis, and outcomes from testicular sperm aspiration. This thorough exploration aims to provide a thorough understanding of our innovative surgical technique and its application in addressing complex cases of obstructive azoospermia.

**Results:**

The procedure was started on the right, the vas deferens was identified and transected. The abdominal side of the vas was intubated and a vasogram performed, there was no obstruction. There was no fluid visible from the testicular side of the vas for analysis, thus we proceeded with VE. Upon inspection of the epididymis dilated tubules were identified. After selecting a tubule for VE, two 10-0 nylon sutures were placed, and it was incised. Upon inspection of the fluid motile sperm was identified. After VE, we performed a novel epididymal occlusion stitch technique. This was completed distal to the anastomosis by placing a 7-0 prolene through the tunica of the epididymis from the medial to lateral side. This stitch was then tightened down with the goal to largely occlude the epididymis so that sperm will preferentially travel through the anastomosis. The steps were then repeated on the left. At 3-month follow up, the patient had no change in testicular size as compared with preoperative size (18cc), he had no testicular or incisional discomfort, and on semen analysis he had presence of motile sperm. After 3 months post-surgery, the patient had motile sperm seen on semen analysis.

**Discussion:**

The introduction of a novel epididymal occlusion stitch demonstrates a targeted strategy to enhance the success of microscopic VE. Encouragingly, a 3-month post-surgery follow-up reveals the presence of motile sperm, reinforcing the potential efficacy of our approach. This is promising given the historical lower patency, delayed time to patency, and higher delayed failure rates that patients who require VE experience (10). In total, 40% of all azoospermia cases can be attributed to obstruction. The conventional treatments for obstructive azoospermia involve microsurgical reconstruction and surgical sperm retrieval followed by IVF. While microsurgical reconstruction has proven to be economically viable, the quest for enhanced success rates has led to the exploration of innovative techniques. Historically, the evolution of VV and VE procedures, initially performed in the early 20th century, laid the foundation for contemporary microsurgical approaches (11). Notably, the microscopic VV demonstrated significant improvements in patency rates and natural pregnancy likelihood, as evidenced by the seminal Vasovastomy Study Group study in 1991 (8). In contemporary literature, success rates particularly for VE remain unchanged for the past three decades since the original published success rates by the Vasectomy Reversal Study Group (12). VE is associated with a longer time to patency as well with patients taking 2.8 to 6.6 months to have sperm return to ejaculate as compared to 1.7 to 4.3 months for those undergoing VV. Additionally, of those patients who successfully have sperm return to the ejaculate after VE up to 50% will have delayed failure compared to 12% for those undergoing VV who are patent. Finally, of those who experience delayed failure after undergoing VE it usually occurs earlier with studies reporting as early as 6 months post-operatively (20). Given the lack of improvement and significantly worsened outcomes with VE further surgical refinement is a constant goal for surgeons performing this procedure. Conclusion: In conclusion, this video is both a demonstration and a call to action for commitment to surgical innovation. We aim to raise the bar in VE success rates, ultimately bringing tangible benefits to patients and contributing to the ongoing evolution of reproductive medicine. The novel epididymal occlusion stitch emerges as a beacon of progress, promising not only enhanced safety but also potential reductions in patency time. Surgical excellence and methodological refinement, as exemplified in this video, lay the foundation for a future where male reproductive surgery continues to break new ground.

Available at: http://www.intbrazjurol.com.br/video-section/20240222_Ramasamy_et_al
